# Norovirus GII.21 in Children with Diarrhea, Bhutan

**DOI:** 10.3201/eid2104.141856

**Published:** 2015-04

**Authors:** Takaaki Yahiro, Sonam Wangchuk, Takeshi Wada, Chimmi Dorji, Takashi Matsumoto, Mimi Lhamo Mynak, Kunzang Pem Tshering, Akira Nishizono, Kamruddin Ahmed

**Affiliations:** Oita University, Yufu, Oita, Japan (T. Yahiro, T. Wada, T. Matsumoto, A. Nishizono, K. Ahmed);; Ministry of Health, Royal Government of Bhutan, Thimphu, Bhutan (S. Wangchuk, C. Dorji);; Jigme Dorji Wangchuk National Referral Hospital, Thimphu (M.L. Mynak);; University of Medical Sciences, Royal Government of Bhutan, Thimphu (K.P. Tshering)

**Keywords:** Norovirus, prevalence, children, diarrhea, Bhutan, viruses, enteric infections

**To the Editor:** Noroviruses are nonenveloped viruses of the family *Caliciviridae* with a single-stranded RNA genome. In developing countries, noroviruses cause >200,000 deaths annually among children <5 years of age (*1*). Noroviruses are divided into 6 genogroups, GI–GVI, which are further divided into genotypes. Twenty-nine of the genotypes cause human infections (*2*). Worldwide, most GII infections are caused by GII.4, followed by GII.3 or GII.6 and then other genotypes, such as GII.2, GII.12, GII.13, GII.17, and GII.7, in varying proportions. We report that GII.21 is the major genotype causing diarrhea in children in Bhutan.

During February 2010–December 2012, fecal samples were collected from children <5 years of age with watery diarrhea who were seen at the outpatient and inpatient clinics of the Department of Pediatrics, Jigme Dorji Wangchuk National Referral Hospital, Thimphu, Bhutan. We extracted RNA from rotavirus-negative fecal samples by using the QIAamp Viral RNA Kit (QIAGEN, Hilden, Germany); norovirus was detected by reverse transcription PCR by amplifying capsid gene at C region (*3*). PCR results were confirmed by nucleotide sequencing of the amplicons (*4*). We determined genogroups and genotypes by submitting nucleotide sequences to the Norovirus Genotyping Tool (http://www.rivm.nl/mpf/norovirus/typingtool).

We collected 15 water samples (Technical Appendix Table), including some from streams and water tanks that are sources of the Thimphu city water supply. Some samples were arbitrarily collected from taps from different locations in Thimphu. All samples were assayed for total coliforms, thermotolerant coliforms, and norovirus (*5*).

We performed a multiple sequence alignment using MUSCLE and conducted the phylogenetic analyses with the neighbor-joining method using MEGAS software (http://www.megasoftware.net). The branching patterns were evaluated statistically on the basis of bootstrap analyses of 1,000 replicates.

We tested 270 samples for norovirus. The mean age of children tested was 13.9 months. Sixty-four (23.7%) children were positive for norovirus. Results were positive for genotype GI in 4/210 samples (0/56 in 2010, 3/123 in 2011, and 1/31 in 2012); all positive samples were from boys in the outpatient clinic. Genotype distribution of norovirus GI was 1 GI.3 in 2012 and 1 GI.1 and 2 GI.9 in 2011.

Sixty of the 270 samples were positive for genotype GII norovirus (17/73 in 2010, 24/147 in 2011, and 19/50 in 2012). Of these, 32 (53.3%) were from boys. Forty-six children were outpatients. Most (85%) of the GII norovirus cases occurred in children <23 months of age; 63.3% of infection occurred in children 6–23 months of age (Technical Appendix Figure 1). By patient age group, the pattern for norovirus GII.21 was similar; 76% of infections occurred in children <23 months, and 62% infections occurred in children 6–23 months.

We determined seasonal distribution of norovirus GII for 52 cases; 21 cases occurred during winter, and only 2 cases occurred during autumn. Nine cases each occurred during spring and summer, and 11 cases occurred during the rainy season (late June through late September). In 2010, GII.3 predominated, followed by GII.4 and GII.7; in 2011, GII.21 was dominant, followed by GII.6, GII.2, and GII.8, then others; and in 2012, GII.21 remained the dominant strain, followed by GII.2, GII.4, and GII.6 (Figure). A Sydney variant of norovirus GII.4 was identified in 2012. Only 1 tap water sample (sample W8) from a house was positive for norovirus and coliform bacteria; and nucleotide sequencing of the norovirus amplicon confirmed it as GII.21. Phylogenetic analysis showed that non-GII.21 genotypes from Bhutan were closely associated with strains from Thailand and Korea (Technical Appendix Figure 2).

**Figure:**
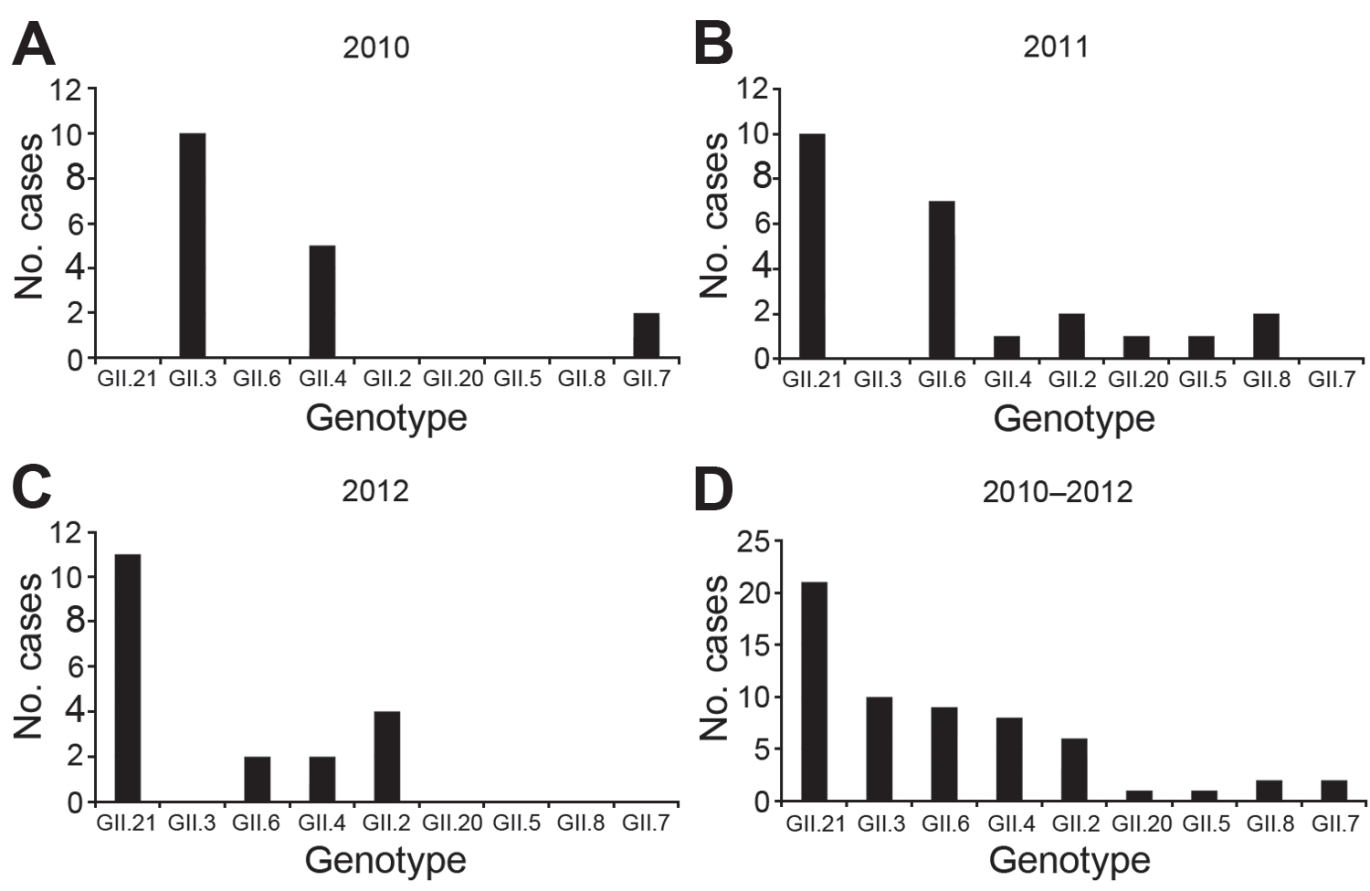
Yearly distributions of different genotypes of norovirus genogroup GII (A–C) and total numbers (D), Bhutan.

In Bhutan, similar to other countries, genogroup GII is mainly responsible for norovirus infections, and most infections occur in younger children (*6*,*7*). In 2010, norovirus GII.3 was the dominant genotype in Thimphu, but in 2011, GII.21 became dominant and continued throughout 2012. GII.21 has been identified mostly in wastewater or rivers, and infections in human have been infrequently attributed to it (*8*). One GII.21 outbreak in a long-term care facility for elderly persons has been reported from the United States (*9*).

Why children in Thimphu were infected by GII.21 is not clear. The detection of norovirus GII.21 in 1 water sample suggests that the source of the outbreak might be tap water; however, GII.21 was not detected in the water related to the tap water supply system. Further examination using repeated samples from different sources is needed. Continuous dominance by GII.21 over 2 years indicates that norovirus of this genotype might have been established in the children of Thimphu, and human-to-human transmission might be ongoing. Determining the environmental source of norovirus GII.21 in Bhutan and developing prevention strategies to control the spread are urgently needed.

Technical Appendix Sample collection date and detailed information about the location, temperature, pH, and fecal coliform count of the sample; age distribution of norovirus GII and GII.21 among children in Thimphu, Bhutan; and phylogenetic tree of the capsid gene at the C region of noroviruses.
