# Supplementary material for: Norovirus GII.21 in Children with Diarrhea, Bhutan
Source: Emerg Infect Dis. 2015 Apr;21(4):732–4. doi: 10.3201/eid2104.141856 (PMC4378497; doi:10.3201/eid2104.141856)
Supplement: Technical Appendix — Sample collection date and detailed information about the location, temperature, pH, and fecal coliform count of the sample; age distribution of norovirus GII and GII.21 among children in Thimphu, Bhutan; and phylogenetic tree of the capsid gene at the C region of noroviruses. [file 14-1856-Techapp-s1.pdf]

Article DOI: http://dx.doi.org/10.3201/eid2104.141856

# Norovirus GII.21 in Children with Diarrhea, Bhutan

## Technical Appendix

Technical Appendix Table. Sample collection date and detailed information of the location, temperature, pH, and fecal coliform count of the samples

| Sample no. | Collection date | Place | Longitude | Latitude | Elevation, m | Water temperature, °C | pH | Fecal CFU/100 mL |
|---|---|---|---|---|---|---|---|---|
| A | 2013/11/21 | Motithang source (Big stream) | 27°28’43.5’’ | 89°35’48.6’’ | 2800 | 4.5 | 8.1 | None |
| B | 2013/11/21 | Motithang source (Small stream) | 27°28’43.7’’ | 89°35’48.5’’ | 2803 | 5 | 7.5 | 3 |
| W1 | 2013/11/22 | Motithang source (Big stream) | 27°28’43.5’’ | 89°35’48.6’’ | 2800 | 4 | 8.2 | None |
| W2 | 2013/11/22 | Motithang source (Small stream) | 27°28’43.7’’ | 89°35’48.5’’ | 2803 | 5.5 | 7.8 | None |
| W3 | 2013/11/22 | Junshina source | 27°30’14.0’’ | 89°38’08.0’’ | 2343 | 5 | 8.2 | None |
| W4 | 2013/11/22 | Cheri side | 27°35’36.4’’ | 89°37’42.9’’ | 2574 | 4.5 | 8.7 | None |
| W5 | 2013/11/22 | Dodena river | 27°35’36.0’’ | 89°37’44.3’’ | 2574 | 4.5 | 8.6 | None |
| W6 | 2013/11/22 | Wangchu river | 27°27’20.1’ | 89°39’06.1’’ | 2310 | 5 | 8.6 | 368 |
| W7 | 2013/11/22 | Simtokha river | 27°26’18.6’’ | 89°40’21.1’’ | 2375 | 5 | 8.5 | 344 |
| W8 | 2013/11/23 | A house in Changjiji Residential Colony | 27°27’14.5’’ | 89°39’13.7’’ | 2284 | 12 | 7.8 | 1 |
| W9 | 2013/11/23 | Changjiji Pump house | 27°27’14.3’’ | 89°39’13.7’’ | 2230 | 12 | 7.7 | None |
| W10 | 2013/11/23 | Changjiji river tank | 27°27’15.9’’ | 89°39’23.2’’ | 2312 | 12.5 | 7.7 | 2 |
| W11 | 2013/11/23 | Lungtenphu Primary School | 27°27’27.8’’ | 89°37’56.1’’ | 2473 | 6 | 8.0 | 2 |
| W12 | 2013/11/23 | Changrigphel Lower Secondary School | 27°26’48.3’’ | 89°39’45.0’’ | 2307 | 10 | 7.9 | 2 |
| W13 | 2013/11/23 | Changzamtok Lower Secondary School | 27°27’42.2’’ | 89°38’25.3’’ | 2346 | 9 | 7.6 | 2 |

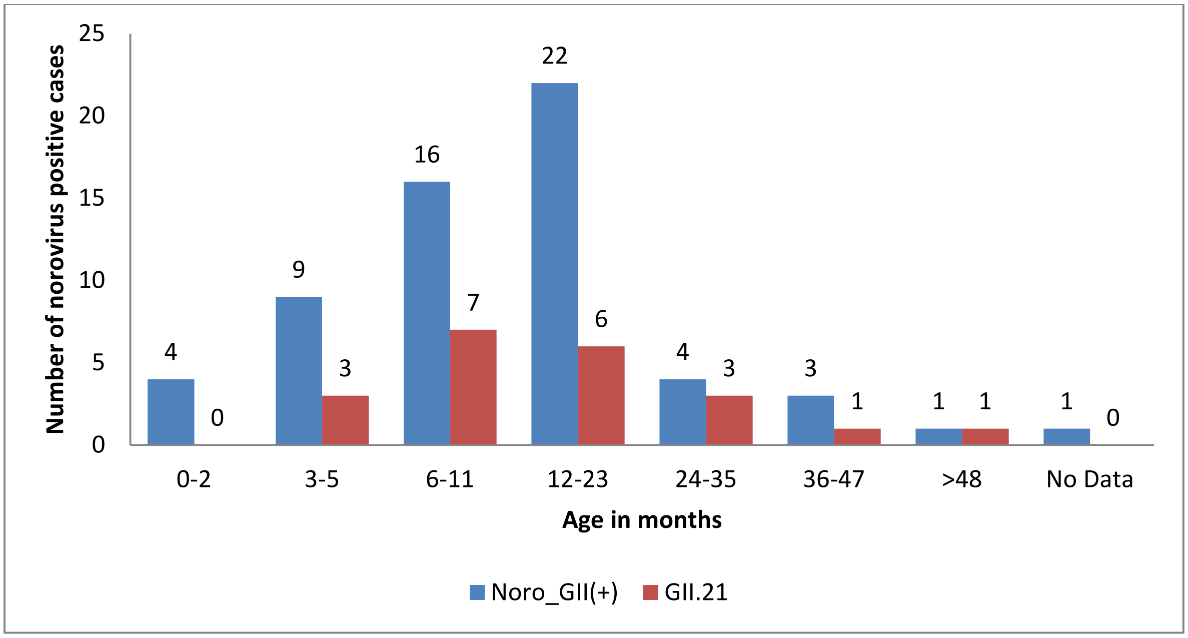


Technical Appendix Figure 1. Age distribution of norovirus (NoV) GII and GII.21 among children in Thimphu, Bhutan. Blue and red columns represent the number of patients with NoV GII and NoV GII.21, respectively among the indicated age categories.

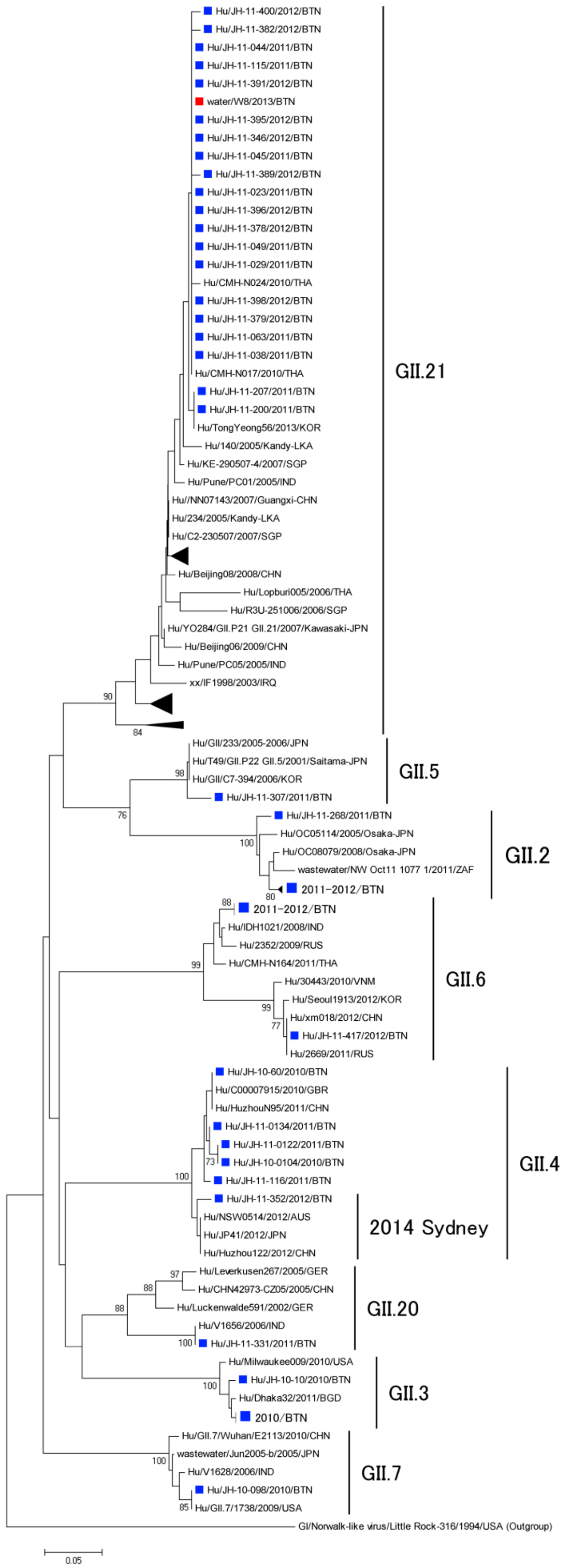
Hu/JH-11-400/2012/BTN
Hu/JH-11-382/2012/BTN
Hu/JH-11-044/2011/BTN
Hu/JH-11-115/2011/BTN
Hu/JH-11-391/2012/BTN
water/W8/2013/BTN
Hu/JH-11-395/2012/BTN
Hu/JH-11-346/2012/BTN
Hu/JH-11-045/2011/BTN
Hu/JH-11-389/2012/BTN
Hu/JH-11-023/2011/BTN
Hu/JH-11-396/2012/BTN
Hu/JH-11-378/2012/BTN
Hu/JH-11-049/2011/BTN
Hu/JH-11-029/2011/BTN
Hu/CMH-N024/2010/THA
Hu/JH-11-398/2012/BTN
Hu/JH-11-379/2012/BTN
Hu/JH-11-063/2011/BTN
Hu/JH-11-038/2011/BTN
Hu/CMH-N017/2010/THA
Hu/JH-11-207/2011/BTN
Hu/JH-11-200/2011/BTN
Hu/TongYeong56/2013/KOR
Hu/140/2005/Kandy-LKA
Hu/KE-290507-4/2007/SGP
Hu/Pune/PC01/2005/IND
Hu//NN07143/2007/Guangxi-CHN
Hu/234/2005/Kandy-LKA
Hu/C2-230507/2007/SGP
Hu/Beijing08/2008/CHN
Hu/Lopburi005/2006/THA
Hu/R3U-251006/2006/SGP
Hu/YO284/GII.P21 GII.21/2007/Kawasaki-JPN
Hu/Beijing06/2009/CHN
Hu/Pune/PC05/2005/IND
xx/IF1998/2003/IRQ
90
84
GII.21
Hu/GII/233/2005-2006/JPN
Hu/T49/GII.P22 GII.5/2001/Saitama-JPN
Hu/GII/C7-394/2006/KOR
Hu/JH-11-307/2011/BTN
98
GII.5
76
Hu/JH-11-268/2011/BTN
Hu/OC05114/2005/Osaka-JPN
Hu/OC08079/2008/Osaka-JPN
wastewater/NW Oct11 1077 1/2011/ZAF
2011-2012/BTN
100
80
GII.2
88
2011-2012/BTN
Hu/IDH1021/2008/IND
Hu/2352/2009/RUS
Hu/CMH-N164/2011/THA
Hu/30443/2010/VNM
Hu/Seoul1913/2012/KOR
Hu/xm018/2012/CHN
Hu/JH-11-417/2012/BTN
Hu/2669/2011/RUS
99
99
77
GII.6
Hu/JH-10-60/2010/BTN
Hu/C00007915/2010/GBR
Hu/HuzhouN95/2011/CHN
Hu/JH-11-0134/2011/BTN
Hu/JH-11-0122/2011/BTN
Hu/JH-10-0104/2010/BTN
Hu/JH-11-116/2011/BTN
Hu/JH-11-352/2012/BTN
Hu/NSW0514/2012/AUS
Hu/JP41/2012/JPN
Hu/Huzhou122/2012/CHN
73
100
2014 Sydney
GII.4
Hu/Leverkusen267/2005/GER
Hu/CHN42973-CZ05/2005/CHN
Hu/Luckenwalde591/2002/GER
Hu/V1656/2006/IND
Hu/JH-11-331/2011/BTN
97
88
88
100
GII.20
Hu/Milwaukee009/2010/USA
Hu/JH-10-10/2010/BTN
Hu/Dhaka32/2011/BGD
2010/BTN
100
GII.3
Hu/GII.7/Wuhan/E2113/2010/CHN
wastewater/Jun2005-b/2005/JPN
Hu/V1628/2006/IND
Hu/JH-10-098/2010/BTN
Hu/GII.7/1738/2009/USA
100
85
GII.7
GI/Norwalk-like virus/Little Rock-316/1994/USA (Outgroup)
0.05

Technical Appendix Figure 2. Phylogenetic tree of the capsid gene at the C region of noroviruses constructed by using nucleotide sequence by neighbor-joining method. For better view of the tree highly related strains of the following genotypes have been collapsed: GII.2, GII.6, and GII.3 are from Bhutan, and GII.21 is from other countries. Numbers adjacent to the nodes represent the bootstrap values. Values <50% are not shown. Each strain is identified by the source of detection followed by strain number, country of origin, and year of detection. Bhutan strains from humans and water sample are indicated by blue and red filled square, respectively. The DDBJ/EMBL/GenBank accession numbers for Bhutan strains are as follows: LC009578–LC009630. Scale bar indicates nucleic acid substitutions per site.
